# Effects of overexpression of IL-10, IL-12, TGF-β and IL-4 on allergen induced change in bronchial responsiveness

**DOI:** 10.1186/1465-9921-7-72

**Published:** 2006-05-08

**Authors:** Chi-Ling Fu, Yi-Ling Ye, Yueh-Lun Lee, Bor-Luen Chiang

**Affiliations:** 1 Graduate Institute of Immunology, College of Medicine, National Taiwan University, Taiwan, Republic of China; 2 Department of Microbiology and Immunology, Taipei Medical University, Taiwan, Republic of China; 3 Department of Pediatrics, National Taiwan University Hospital, Taiwan, Republic of China

## Abstract

**Background:**

An increasing prevalence of allergic diseases, such as atopic dermatitis, allergic rhinitis and bronchial asthma, has been noted worldwide. Allergic asthma strongly correlates with airway inflammation caused by the unregulated production of cytokines secreted by allergen-specific type-2 T helper (Th2) cells. This study aims to explore the therapeutic effect of the airway gene transfer of IL-12, IL-10 and TGF-β on airway inflammation in a mouse model of allergic asthma.

**Methods:**

BALB/c mice were sensitized to ovalbumin (OVA) by intraperitoneal injections with OVA and challenged by nebulized OVA. Different cytokine gene plasmids or non-coding vector plasmids were instilled daily into the trachea up to one day before the inhalatory OVA challenge phase.

**Results:**

Intratracheal administration of IL-10, IL-12 or TGF-β can efficiently inhibit antigen-induced airway hyper-responsiveness and is able to largely significantly lower the number of eosinophils and neutrophils in bronchoalveolar lavage fluid of ovalbumin (OVA) sensitized and challenged mice during the effector phase. Furthermore, the effect of IL-10 plasmids is more remarkable than any other cytokine gene plasmid. On the other hand, local administration of IL-4 gene plasmids before antigen challenge can induce severe airway hyper-responsiveness (AHR) and airway eosinophilia.

**Conclusion:**

Our data demonstrated that anti- inflammatory cytokines, particularly IL-10, have the therapeutic potential for the alleviation of airway inflammation in murine model of asthma.

## Background

Asthma is an immunological disease that has increased dramatically in prevalence over the past two decades. It is characterized by airway hyper-reactivity to a variety of specific and non-specific stimuli, severe chronic airway inflammation with pulmonary eosinophils, mucus hypersecretion, and increased serum IgE levels. Activation of Th2 cells in the respiratory tract is now believed to be responsible, in part, for the pathogenesis of this disease. Th2 cells secreting IL-4, IL-5, and IL-13 have been identified in the airways of asthmatics [1]. Th2 cytokines produced in the respiratory tract, airway eosinophilia, high levels of serum IgE, and mast cell activation [2,3], are all believed to contribute to the pathological consequences inducing airway hyper-responsiveness (AHR), epithelial damage, and mucus hypersecretion.

Whereas the immunological mechanisms that induce asthma and allergies are relatively well characterized, the specific mechanisms that transpire *in vivo *to downmodulate Th2 cell-mediated allergic inflammatory responses are not yet clear. The Th1-relatived cytokines, such as IL-12 and IFN-γ, are the candidate cytokines for the treatment of allergic diseases as they downregulate Th2 responses [4]. There is strong evidence regarding the therapeutic effect of Th1 cytokine administration. Using Th1-related cytokine proteins [5-7] and constructed plasmids expressing cytokine genes [8-10], airway inflammation could be decreased. According to our previous study [11], we also demonstrated that the local transfer of the IL-12 gene to the respiratory tract could modify allergic inflammation and airway hyper-responsiveness (AHR). However, recent studies have shown that not only Th1-related cytokines, but also other anti-inflammatory cytokines, including TGF-β and IL-10, can downregulate Th2 responses and might also play an important role in regulating pulmonary inflammation and asthma [12,13]. IL-10 and TGF-β, which are pleiotropic cytokines with significant anti-inflammatory and immunosuppressive properties, are key regulators in the maintenance of immunological homeostasis. In humans, relative underproduction of IL-10 by alveolar macrophages and in the sputum of patients with asthma has been reported [14,15], which suggests an essential role IL-10 in regulating airway inflammation. In addition, TGF-β inhibits the production of proinflammatory cytokines from macrophages, B cells, and T cells and is a potent inhibitor of T cell-mediated immune responses, both *in vitro *[16,17] and *in vivo *[18,19]. Moreover, TGF-β has been postulated in the mechanism of oral tolerance, which is mediated by regulatory T cells that produce TGF-β preferentially induced at mucosal sites, possibly under the influence of IL-10 and/or IL-4 [20]. Recently, Hansen et al. showed that not only TGF-β-producing T cells [21] but also IL-10-producing T cells [22] could abolish AHR and airway inflammation in a murine model of asthma. Thus, not only Th1-related cytokine but also anti-inflammatory cytokines can regulate airway inflammation. However, the different effects between these cytokines on alleviating airway inflammation still need further investigation. The purpose of the current study was to compare the effect of four different cytokine genes plasmid including IL-12, IL-10, and TGF-β on the effector phase of allergen-induced AHR and airway eosinophilic inflammation.

It is reported that eosinophils are so important in the asthma, because the toxic products in its granules were proven to directly damage lung tissue [23]. Amongst eosinophil-active chemoattractants, eotaxin has also been demonstrated to selectively induce eosinophil recruitment to the airway undergoing allergic reaction [24,25]. In addition, both leukotriene B4 (LTB4) and prostaglandin E2 (PGE2) are potent pro- inflammatory mediators and are involved in several inflammatory diseases [26]. In this current study, we have compared the levels of eotaxin, LTB4 and PGE2 in the BALF to investigate the role of cytokine gene in regulating the production of these inflammatory mediators and try to address possible mechanisms for the effect of different cytokine genes.

## Methods

### Animals

Female BALB/c mice were obtained from and maintained at the Animal Center of the College of Medicine of National Taiwan University. Animals were used between 6 and 10 weeks of age and were age-matched within each experiment. The animal study protocol was approved by the committee of College of Medicine, National Taiwan University.

### Plasmids and preparation of lipid-plasmid DNA complexes

For the construction of plasmid DNA encoding murine IL-10 or TGF-β, the cDNA for murine IL-10, or TGF-β was cloned by reverse transcription- polymerase chain reaction (RT-PCR) from normal mouse spleen cells, using primers based on the published cytokine sequence. The cDNA was sequenced and *in vitro *expression was confirmed by enzyme-linked immunosorbent assay (ELISA) and bioassay (data not shown). The cytokine gene expression vector utilized the human cytomegalovirus (CMV) immediate-early promoter and the simian virus 40 (SV40) polyadenylation sequence. The vector without a gene insert (empty vector) served as a control for *in vivo *gene delivery studies.

The construction of pscIL-12 vectors has been described previously [27]. Briefly, a linker of 54-bp in length in the pscIL-12 plasmids connected the p40 and p35 subunits of the murine IL-12 gene. The p40 and p35 subunits were obtained by polymerase chain reaction (PCR) from the BLpSV35 and BLpSV40 plasmids. Recombinant PCR, using the p40 and P35 PCR products as the DNA templates and the *Sal *I-containing and the *Bam *HI-containing primers as such primers, generated the single- chain IL-12 genes. The resulting recombinant PCR fragments were cloned at the *Sal *I and *Bam *HI sites of the pCMV vector. Plasmid DNA was subsequently introduced into the *Escherichia coli *DH5α by transformation. The plasmids were purified using EndoFree plasmid kits (QIAGEN, Valencia, CA) and suitable for gene therapy.

For intra-tracheal delivery, lipid-DNA complexes were prepared by combining 15 μl lipofectAMINE (Life Technologies, Gaithersburg, MD), per 10 μg of plasmid DNA at a final volume of 15 μl in PBS. The expression of cytokine plasmid in pulmonary tissues was determined by the cytokine ELISA of BALF collected 48 hr post- injection [data not shown, [27]].

### Administration of DNA-lipid complexes

Intra-tracheal administration was accomplished by the use of a No. 23 steel gavage tube and a 1.0-ml syringe. Animals were anesthetized (pentobarbital sodium salt, Tokyo Chemical Industry, Tokyo, Japan, 10 mg/ml solution, 0.005 ml/g body weight) prior to intra-tracheal injection and placed in dorsal recumbence on an inclined board. The gavage tube was directed into the proximal trachea, and then the lipid-DNA solution was slowly injected. Proper positioning of the tube was assured by visualization of movement of the fluid meniscus and by palpation of the gavage tube moving across the tracheal rings. A volume of 30 μl lipid-DNA mixture was injected intra-tracheally, such that each mouse received 10 μg of plasmid DNA. This technique works well without involving any surgical procedure and allows the aspirated material to spread over the whole lung.

### Administration of cytokine plasmid into allergen-sensitized mice

**Figure 1:**
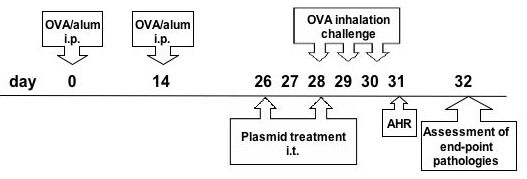
**Treatment regimen**. Time line representation of the OVA protocol used and the intratracheal injection of cytokine plasmid. i.p., intraperitoneal; i.t., intra-tracheal.

BALB/c mice were sensitized by an intraperitoneal injection with OVA (Sigma, St. Louis, MO, 10 μg) complexed with aluminum potassium sulfate (Imject Alum, Pierce Biotechnology Inc., Rockford, IL, 2 mg) on day 0. On day 14, the mice were boosted with OVA (30 μg) adsorbed to alum. As the negative control group, the mice were injected with PBS only. To examine the therapeutic effects of different cytokine plasmids, each group of mice received intra-tracheal delivery of 10 μg pCDNA vector only or a single chain IL-12 DNA plasmid or TGF-β plasmid or IL-10 plasmid liposome complexes, respectively, two days before the inhalation challenge on day 26 and 28. On day 29, and 30, mice were challenged with OVA (100 μg in a total volume 40 μl) by intranasal administration on consecutive days (Fig. 1).

In order to test varying doses of each a single dose of a cytokine gene, some mice received 2.5 μg IL-10 gene plasmid liposome complex (p-IL-10-low) or 20 μg IL-10 gene plasmid liposome complex (p-IL-10-hi). In the cytokine gene combination experiment, some mice received 10 μg IL-10 gene plasmid plus 10 μg single-chain IL-12 gene plasmid at a final volume of 30 μl DNA-liposome complex (pIL-10 + pscIL-12).

### Measurement of airway hyper-responsivenes

Airway responsiveness was measured as a change in function after challenge with aerosolized mechacholine (Mch) in conscious, spontaneously breathing animals by barometric plethysmography (Buxco, Troy, NY) as described in the literature [28]. Pressure differences were measured between the main chamber of the plethysmograph, containing the animal and a reference chamber (box pressure signal). Mice were challenged with aerosolized saline (for the baseline measurement) or Mch (6.25 to 50 mg/ml) for three minutes and readings were taken and averaged for three minutes after nebulization. The Penh value for each minute was recorded and after the third recorded value, the average Penh value was divided by the Penh of normal saline and was presented as a relative percentage increase of Penh.

### Analysis of bronchoalveolar lavage (BAL) fluid and lung histology

At 48 hours after the last aerosol exposure, all groups of mice were bled from the retro-orbital venous plexus and terminated. The lungs were immediately lavaged via the tracheal cannula with 3 × 1 ml of HBSS, free of ionized calcium and magnesium. The lavage fluid was centrifuged at 400 × g for 10 minutes at 4°C. After washing, the cells were resuspended in 1 ml HBSS, and total cells counts were determined by counting in a hemocytometer. Cyto- centrifuged preparations were stained with Liu's stain for different cell counts. A minimum of 200 cells were counted and classified as macrophages, lymphocytes, neutrophils, and eosinophils, based on standard morphological criteria.

After the lavage, the lungs were immediately removed and fixed in 10% neutral- buffered formalin, routinely processed, and embedded in paraffin wax. Five-micrometer sections were prepared and stained with hematoxylin and eosin (H&E).

### Eotaxin level in bronchoalveolar lavage

The concentration of eotaxin was assayed with an ELISA kit (R&D Systems Inc., Minneapolis, MN) according to the manufacturer's instructions. Briefly, the bronchoalveolar lavage of each condition was added to wells precoated over- night at 4°C with anti-eotaxin antibody. After two hours of incubation, the plates were washed and biotin-conjugated antibody was added. After two more hours at room temperature, HRP-avidin was then added, and the OD (at 450 nm) values were converted to concentrations of chemokine in the BALF. The sensitivity of this assay was 1.9 pg/ml for eotaxin.

### Measurement of cytokines

Quantifications of IL-10, IL-12, and TGF-β in the BAL fluids were evaluated using commercially available ELISA kits (Duoset, R & D, Minneapolis, MN, USA). Briefly, the BAL fluids were added to wells pre-coated over night at 4°C with anti-cytokine Ab. After 2 hours of incubation, the plates were washed and biotin-conjugated Ab was added. After two more hours at room temperature, HRP-avidin was added to each well. The substrate tetramethylbenzidine was then added and the OD (at 450 nm) values were converted to concentrations of cytokines in the BAL fluids. The sensitivity of this assay was 31.3 pg/ml for IL-10, IL-12 and TGF-β.

### Quantification of PGE2 and LTB4

PGE2 or LTB4 levels in the BALF were determined using the PGE2 enzyme immunoassay kit or LTB4 enzyme immunoassay kit (Assay Designs, Inc., Ann Arbor, MI) according to the manufacturer's instructions. The detection limits for PGE2 and LTB4 are 39 and 47 pg/ml, respectively.

### Statistical analysis

Data are expressed as the mean ± SEM for each group. The statistical significance of the differences between various treatment groups was assessed with the Mann-Whitney U test for non-parametric data.

## Results

### The effect of different cytokine genes on methacholine-induced increase in AHR and airway eosinophilia

**Figure 2:**
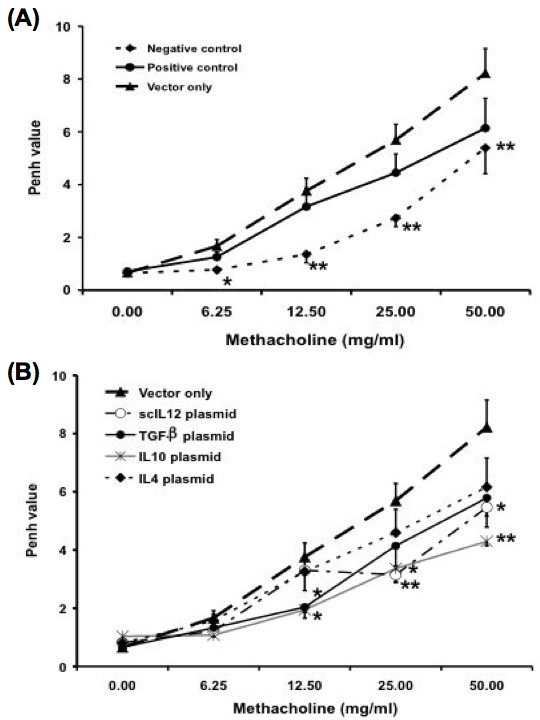
**Effect of different cytokine genes on methacholine- induced increases in airway hyperresponsiveness (AHR)**. Mice were treated as described in Figure 1. One day after the last OVA challenge, AHR was measured in response to increasing concentrations of methacholine (0–50 mg/ml) in conscious mice placed in a whole-body plethysmograph. "Negative control" mice were mice that were sensitized and challenged with normal saline. Both "positive control" mice and "vector-only" mice were mice that immunized and challenged with OVA. However, only the "vector-only" group was treated with mock DNA plasmid. Data are representative of three separate experiments with similar results. The columns and error bars represent mean ± SEM for each group. * P < 0.05, ** P <0.01 as compared with the vector-only treated control group

In order to examine the effect of different cytokine genes, lipid- plasmid DNA complexes were administered intratracheally 48 hours prior to OVA challenge in OVA-sensitized mice. One day after the last allergen challenge, each group of mice was measured for airway responsiveness to aerosolized methacholine (Figure 2). We measured the extent of airway constriction of mice using the Buxco system. The Penh (pause of enhance) increased as the concentration of methacholine increased. The mice sensitized with OVA but only administered mock vector-only developed marked increased airway responsiveness to methacholine challenge compared with mice challenged without prior sensitization. We also immunized the mice with OVA only without any delivery of DNA plasmid as the control. Actually the severity of airway inflammation was very similar between these two groups.

To further assay the cytokine levels in BAL fluids (BALFs) of mice received cytokine genes treatment. BALFs collected from control and cytokine gene-treated mice were analyzed with sandwich-ELISA. The results showed that the level of IL-12 (365.0 ± 111.9 pg/ml vs. 85.7 ± 16.1 pg/ml), IL-10 (453.6 ± 99.2 pg/ml vs. 66.4 ± 22.6 pg/ml) and TGF-β (1110.6 ± 47.2 pg/ml vs. 166.0 ± 25.5 pg/ml) increased in individual cytokine gene delivered mice compared to the control mice respectively.

Similar to our previous study [11], local administration of single- chain IL-12 gene plasmids exerted the therapeutic effect in OVA-induced asthma model as in the Der p 1-induced asthma model. Further, administration of TGF-β gene plasmids and IL-10 gene plasmids has also been found to inhibit the increase in airway responsiveness to methacholine after aerosol challenge in OVA-sensitized mice when compared with that of the mock vector-only group.

**Figure 3:**
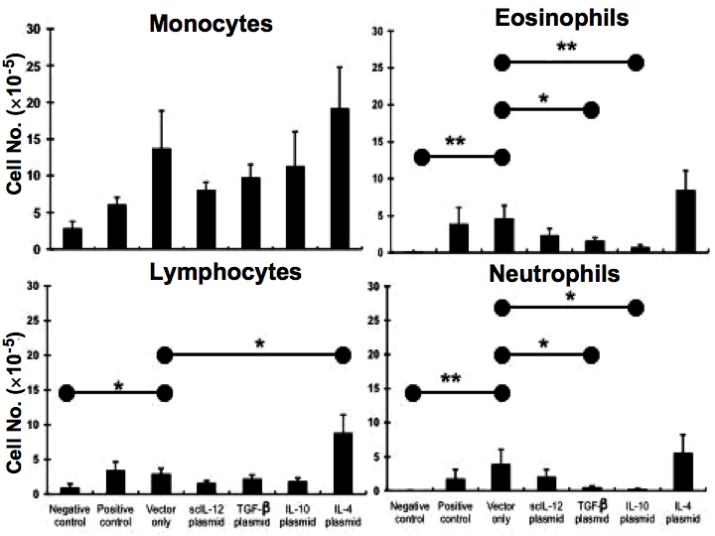
**Effects of different cytokine gene plasmids on airway eosinophilic inflammation in mice after aerosol challenge**. Mice were treated as described in Figure 1. Two days after the last OVA challenge, mice were sacrificed, and the bronchoalveolar lavage fluid (BALF) was collected. The cell compositions in BALF of different groups of mice were analyzed. Data are representative of three separate experiments with similar results. The columns and error bars represent mean ± SEM for each group. * P < 0.05, ** P < 0.01 as compared with the vector-only treated control group

Further, we analyzed the cellular composition in the BAL fluid of sensitized mice 48 hours after the last challenge to determine whether the local transfer of cytokine gene plasmids could alleviate airway inflammation. In positive control group mice, exposure to aerosolized OVA often induced a marked increase in the number of neutrophils and eosinophils in BALF (Figure 3). In contrast, a few cells were noted in non-sensitized mice. The delivery of vector-only plasmid did not decrease the airway inflammation in murine model of asthma. However, administration of scIL-12-encoding vector partially decreased the recruitment of eosinophils (p = 0.12) compared to the vector-only treated group. A similar result was also found in mice treated with TGF-β and IL-10-encoding vector, although a certain degree of variance was noted. Administration of IL-10 gene plasmids (p = 0.009) had a more significant decrease in the level of eosinophilia than those given TGF-β (p = 0.04) and IL-12 (p = 0.12) encoding vector. Furthermore, the recruitment of neutrophils was almost completely inhibited by the treatment of IL-10 encoding vector (p = 0.019).

**Figure 4:**
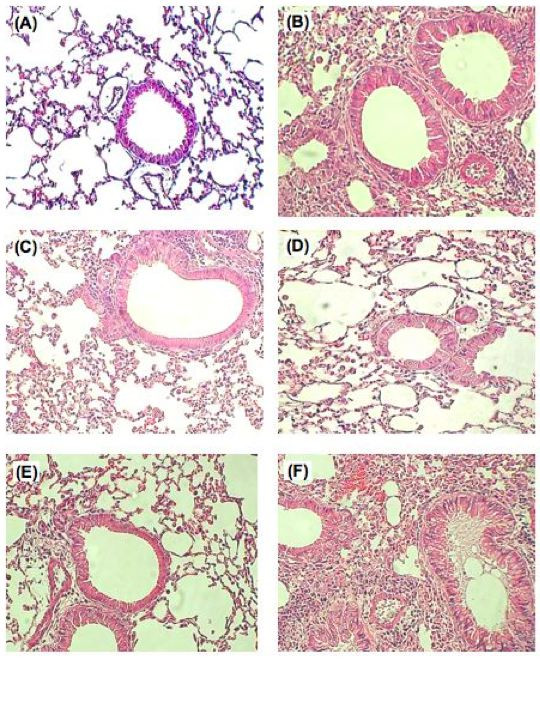
**Histological studies of the lungs of immunized mice with different cytokine gene plasmid treatments**. Mice that had been sensitized and repeatedly challenged with nebulized saline (A) or OVA (B-E) were gavaged with non-coding vector, scIL-12, TGF-β, or IL-10 gene plasmids before the challenge phase. The data showed extensive cellular infiltration of the peri- airway region from vector DNA treated mice (B). In contrast, lung tissue from scIL-12 plasmid treated mice (C), TGF-β plasmid treated mice (D), and IL-10 plasmid treated mice (E) showed a much less severe inflammation histologically. Microscopic images were made with an Olympus microscope at a magnification of 100, and images were representative of the experimental group. Paraffin embedded sections were stained with hematoxylin and eosin.

Histopathologically, many cells infiltrated around the bronchial and lung alveoli in both the control (data not shown) and vector treated group (Fig. 4B); in the contrast, the damage and infiltrative cells were less severe in the scIL-12 plasmid (Fig. 4C) or TGF-β plasmid (Fig. 4D) or IL-10 plasmid treated group (Fig. 4E). These results demonstrated that intratracheal delivery with scIL-12 plasmid; TGF-β plasmid or IL-10 plasmid could efficiently inhibit the infiltration of the cells and reduce the pathological damage within the lung in this mouse model.

We also examined whether the level of OVA-specific serum antibodies were affected by the treatment of different cytokine gene plasmids (data not shown). Ovalbumin-sensitized mice had increased total serum IgE concentrations and produced OVA-specific IgE and IgG1 antibodies after airway challenge with OVA. However, only low levels of OVA-specific IgG2a were detected in serum. Intra-tracheal administration of mock vector DNA did not change OVA-specific antibody levels. The OVA-specific IgE concentrations were also increased, but the increase was not significant. Furthermore, administration of scIL-12, IL-10, or TGF-β plasmid DNA did not significantly change OVA-specific IgE, IgG1, or IgG2a levels in serum.

### The effect of different cytokine gene plasmids on eotaxin and leukotriene B4 (LTB4) levels in BAL fluid

**Figure 5:**
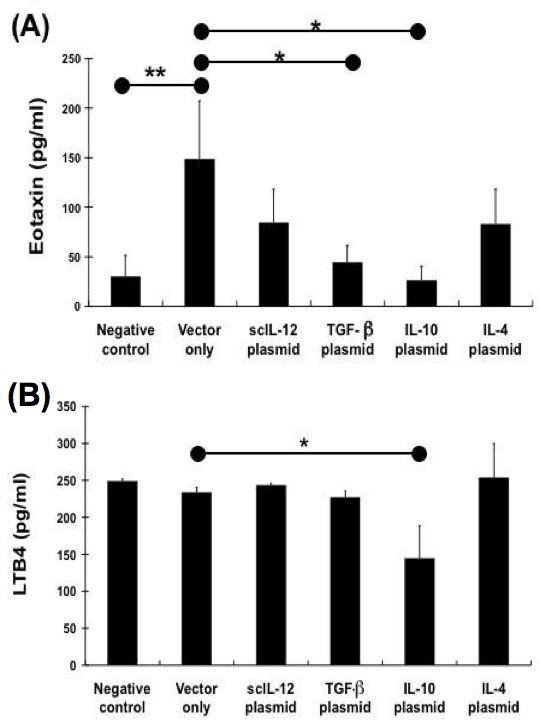
**Effect of different cytokine gene plasmids on eotaxin and LTB4 levels in BAL fluid from mice after aerosol challenge**. Two days after the last OVA challenge, mice were sacrificed, and the BAL fluid was collected. Eotaxin levels in BALF of different groups of mice were measured by ELISA. Data are representative of three separate experiments with similar results. The columns and error bars represent mean ± SEM for each group. * P < 0.05, ** P <0.01 vs. vector-only treated control group

In order to investigate the effects and underlying mechanism(s) of the action of different cytokine gene plasmids on eosinophils recruitment, the inflammatory mediators implicated in regulating eosinophils accumulation was also determined. Allergen challenge via the airway in sensitized mice resulted in a sharp increase in eotaxin levels in BALF (P = 0.005, compared with the negative control). In our previous *in vitro *study, Ye et al. [29] have demonstrated that IL-4 could stimulate lung cells to secret eotaxin, but IL-12 could suppress eotaxin secretion from IL-13 or IL-4 stimulated primary lung cell culture. In present study, *in vivo *experiment also supported this result. Administration of scIL-12 gene plasmid could decrease the level of eotaxin in the BALF. Furthermore, the eotaxin levels in BAL fluid significantly decreased through the delivery of IL-10 (P = 0.019) and TGF-β encoding vector (P = 0.007) in OVA- sensitized mice (Figure 5). The data showed that the eotaxin levels correlate with the reduction in eosinophils in BALF.

LTB4 and PGE2 are potent eicosanoid lipid mediators that are involved in numerous homeostatic biological functions and inflammation [26]. The interaction between eicosanoid may represent means to regulate the release of inflammatory mediators, and may be important for the regulation of cell functions and inflammatory disorders, such as allergic asthma. Previous studies have reported that PGE2 could enhance the production of endogenous IL-10, which inhibits LTB4 production. In this study, the levels of LTB4 and PGE2 in the BAL fluid were also determined after administration of different cytokine gene plasmid. The level of LTB4 and PGE2 in BAL fluid did not show a significant difference among groups treated with different cytokine gene plasmids. However, LTB4 concentrations in the BAL fluid of the IL-10 gene-treated group was obviously lower than that of vector-only treated group (p = 0.085). This result was proven that IL-10 gene plasmid could decrease the production of LTB4 as previous study.

### Dose-dependent effect of IL-10 gene plasmid in the suppression of AHR and airway eosinophilic inflammation in OVA-sensitized mice

**Figure 6:**
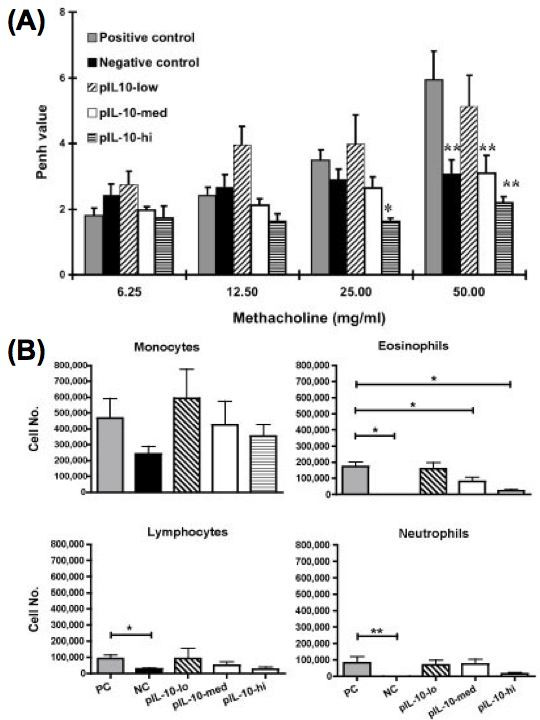
**Intra-tracheal delivery of IL-10 gene plasmid can suppress AHR and airway eosinophilic inflammation in OVA-sensitized mice in a dose-dependent manner**. Mice were sensitized and boosted as described in Fig. 1. On day 27 and 28, some mice received different doses of IL-10 gene plasmid DNA liposome complex by intra-tracheal injection (pIL-10-low: 2.5 μg; pIL-10-med: 10 μg; pIL-10-hi: 20 μg). Then, mice were challenged with 100 μg OVA by intranasal administration on day 29, and 30. On day 31, mice were analyzed. (A) AHR to Mch was measured as described in Material and Methods (n= 4–7 per group). The columns and error bars represent mean ± SEM for each group. * P < 0.05, ** P <0.01 compared with the value of positive controlgroup. (B) Bronchoalveolar lavage fluid (BALF) was collected two days after the last OVA challenge of each group of mice (n = 4–7). The cell compositions in BALF were analyzed. The columns and error bars represent mean ± SEM for each group.

We next decided to investigate the relative efficacy of varying doses of IL-10 gene plasmid for the alleviating effect of the severity of asthma symptom. Mice were sensitized and boosted as previous experiment. On day 27 and 28, some mice received different doses of IL-10 gene plasmid liposome complex by intra-tracheal injection before the last challenge. The results of experiments are shown in Figure 6. It is apparent that the immune- modulating efficacy is correlated with the administrated dose of IL-10 plasmid. Intra-tracheal delivery of related less amount of IL-10 plasmid did not have any effect on the suppression of AHR and airway eosinophils recruitment. However, in mice that received the same amount of IL-10 gene plasmid as above in pIL-10-med group, the severity of airway hyper- responsiveness (p = 0.0022) and eosinophilia (p = 0.026) was significantly decreased. Moreover, while the administration dose was 2-fold amount, the suppressive effect of IL-10 gene plasmid was markedly increased. In pIL-10-hi group mice, high dose IL-10 gene delivery almost completely diminished the eosinophil number in BALF (p= 0.0179) and AHR to methacholine was also decreased (p = 0.0179, compared to the positive control group). These results indicated that *in vivo *IL-10 gene delivery suppressed Ag- induced eosinophilic airway inflammation and AHR in a dose-dependent manner.

### Combination effect of IL-10 and IL-12 gene plasmid in the suppression of AHR and airway eosinophilic inflammation in OVA-sensitized mice

**Figure 7:**
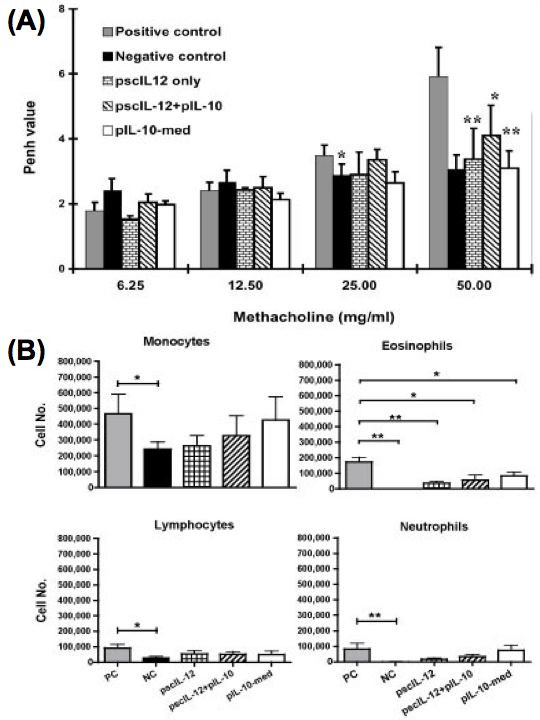
**Comparison of the effect of combined administration of IL-10 gene plasmid and IL-12 gene plasmid to the individual treatment groups on AHR and airway eosinophilic inflammation in OVA-sensitized mice**. Mice were sensitized and boosted as described in Fig. 1. On day 27 and 28, some mice received intra-tracheal injection of IL-10 gene plasmid (pIL-10), single-chain IL-12 gene plasmid (pscIL-12), or pIL-10 plus pscIL-12 DNA liposome complex. Then, mice were challenged with 100 μg OVA by intranasal administration on day 29, and 30. On day 31, mice were analyzed. (A) AHR to Mch was measured as described in Material and Methods (n = 5–7 per group). The columns and error bars represent mean ± SEM for each group. * P < 0.05, ** P <0.01 compared with the value of positive controlgroup. (B) Bronchoalveolar lavage fluid (BALF) was collected two days after the last OVA challenge of each group of mice (n = 5–7). The cell compositions in BALF were analyzed. The columns and error bars represent mean ± SEM for each group. * P < 0.05, ** P < 0.01 as compared with the positive control group

As described above, not only scIL-12 plasmid but also IL-10 plasmid could efficiently inhibit the infiltration of the inflammatory cells and reduce the pathological damage within the lung though intra-tracheal gene delivery. However, it has been reported that IL-10 can inhibit Th1cytokine production via the suppression of IL-12 synthesis in accessory cells [30]. In our present study, we examined the effect of IL-10 gene plasmid and single chain IL-12 gene plasmid, alone or together on the modulation of the airway inflammation of OVA- sensitized mice. As shown in Fig. 7A, AHR to Mch was significantly decreased in mice treated with pIL-10 (p = 0.0022) or psc-IL-12 alone (p = 0.0476). Furthermore, the recruitment of eosinophils in the BALF was also inhibited in both pIL-10 (p = 0.026) and pscIL-12-treated mice (p = 0.0079) (Fig. 7B). These results were similar to our previous experiment. The combination treatment of IL-10 gene and single-chain IL-12 gene plasmid also suppress the airway eosinophilic inflammation (p = 0.0278) (Fig. 8B). However, the effect on the suppression of AHR was not as efficient as the mice which received IL-10 gene plasmid or single-chain IL-12 gene plasmid alone.

## Discussion

Allergic diseases are characterized by the presence of Th2 cells and related cytokines, such as interleukin-4 (IL-4), IL-5, IL-9, and IL-13 with the subsequent development of eosinophils infiltration and chronic inflammation. Although the immunologic mechanisms that induce asthma and allergic diseases are relatively well characterized, the specific mechanisms that transpire *in vivo *to down-modulate Th2-mediated allergic inflammatory responses are yet to be clarified. However, blocking the release or effects of pro-inflammatory cytokines in allergic asthma has provided the basis for the development of novel treatments [12]. In this study, we employed a liposome-mediated genetic transfer approach to examine the therapeutic efficacy of the local pulmonary delivery of various cytokine gene plasmids in the same murine model of asthma in OVA-sensitized mice.

First, we demonstrated that intra-tracheal delivery with scIL-12 plasmid; TGF-β plasmid or IL-10 plasmid could suppress Ag-induced eosinophilic airway inflammation and airway hyper- responsiveness during Ag challenge, an effector phase of the immune response (Fig. 1 and 2). On the other hand, administration of IL-4 plasmid enhanced the severity of airway inflammation.

It is complex in the control of allergic inflammation and asthma, which are involving several different mechanisms and several different cell types and cytokines. Nevertheless, several studies have demonstrated that IL-12 protein can decrease allergen-specific IgE and eosinophils infiltration in a mouse model of airway inflammation [5,6,9,31]. Previous studies have shown that intravenous injection of single chain IL-12 DNA plasmids mixed with liposome achieved the highest protein expression in the lungs and can alleviate airway hyper-responsiveness in an animal model of asthma [32]. Furthermore, the local IL-12 gene transfer to the lung before the tracheal allergen challenge resulted in a remarked decrease in IL-5 levels and a similarly marked increase in IFN-γ, this being consistent with a shift from a Th2 to a Th1 profile [11]. The results of our present study also support the finding that intra-tracheal delivery of IL-12 encoding DNA plasmids can decrease eosinophils infiltration in a murine model of airway inflammation.

Both TGF-β and IL-10 are pleiotropic cytokine with significant anti-inflammatory and immuno-modulatory properties. Thus, we investigated the suppressive effect of these two cytokines in modulating pulmonary inflammation and asthma. TGF-β is a key immunoregulatory factor in the development of unresponsiveness to antigens in the gastrointestinal tract. TGF-β inhibits the production of proinflammatory cytokines from macrophages, B cells, and T cells, and is a potent inhibitor of T cell-mediated immune responses both *in vitro *[17,18] and *in vivo *[19,20]. Administration of TGF-β diminishes the severity of autoimmune diseases, such as collagen-induced arthritis [33], allergic encephalomyelitis (EAE) [19], and experimental colitis [34], and neutralization of TGF-β adversely affects the course of the diseases. Hansen and his colleagues [21] have demonstrated that CD4^+ ^T helper cells engineered to produce TGF-β1 in the respiratory mucosa can indeed reverse allergen-induced airway hyper-reactivity and inflammation. TGF-β- secreting T cells, called Th3 cells, have been shown to play a regulatory role at mucosal sites- e.g., in the induction of oral tolerance [35]. In addition, TGF-β- secreting T cells might also play a significant role in modulating allergic inflammation [21]. However, TGF-β is also a potent inducer of myofibroblasts and collagen synthesis. It has been reported that eosinophils might produce TGF-β to prevent allergen-induced AHR in late phase [36]. In our study, the result has shown that administration of TGF-β plasmid in OVA-sensitized and challenged mice can decrease airway hyper-reactivity, eosinophilia and neutrophilia. In conclusion, TGF-β may plays a role in immuno-regulation, wound healing, and to shorten the inflammatory response when it is applied in the treatment of allergen-induced asthma.

IL-10 can down-regulate cytokine production not only from Th1 cells [37] but also from Th2 cells [38]. Borish et al. [14] reported that asthmatics had a comparatively decreased ability to produce IL-10 by BALF cells and mononuclear cells. These associations led to the speculation that constitutive expression of IL-10 in the airway might contribute to maintain the normal state of allergen nonresponsiveness. However, the role of IL-10 in regulating Th2-mediated diseases such as asthma is controversial. Some reports have indicated that IL-10-deficient mice exhibit exaggerated eosinophilic airway inflammation provoked by a systemic sensitization to Ag and local inhalation of Ag [39,40], whereas another report indicated that IL-10-deficient mice show diminished eosinophilic airway inflammation [41]. Indeed, IL-10 is required for the development of AHR and administration of IL-10 enhanced AHR, though it reduces eosinophilia [42]. The overall physiologic effect of IL-10 is to decrease inflammation. It accomplishes this role primarily by down-regulating synthesis of a number of cytokines, both Th1- and Th2-associated. The result of our study has shown that administration of IL-10 plasmids in OVA-sensitized and challenged mice can decrease airway hyper-reactivity, including eosinophilia and neutrophilia. These results support the concept that IL-10 plays a regulatory role in allergic asthma. As suggested by the effect of IL-10 on airway eosinophilia, IL-10 appears to play a role in regulating eosinophils recruitment. Our findings of a regulatory role for IL-10 in asthma are strongly consistent with the idea that IL-10 production mediates Ag-specific tolerance that protects against allergic diseases and airway inflammation. For example, regulatory T cells producing IL-10 are thought to develop after successful bee venom-specific immunotherapy [43] and during T-cell tolerance induced with respiratory exposure to antigen [44,45].

Subsequently, we also compared the level of inflammatory mediators in BALF of OVA- sensitized mice among different cytokine gene plasmid treated groups. Amongst eosinophil-active chemoattractants, eotaxin has also been demonstrated to selectively induce eosinophil recruitment to the airway undergoing allergic reaction [46,47]. Eotaxin is expressed in many different tissues, and may therefore regulate allergen-induced homing of eosinophils to the site of inflammation. In current study, we found that eotaxin levels in BAL fluid were significantly decreased by treatment with IL-10 or TGF-β encoding vector. It is possible that IL-10 and TGF-β can suppress OVA-induced airway eosinophils recruitment directly by down-regulating the production of eotaxin. Although, the eotaxin level in BALF was not so markedly reduced in the group of IL-12 gene plasmid treated mice (Fig. 5). However, it has been reported that IL-12 in regulating eosinophil function by increasing eosinophil apoptosis. On the other hand, we should consider whether the dose of IL-12 gene plasmid affects the efficiency of IL-12 gene plasmid in the inhibiting the airway inflammation.

Moreover, we also detected the level of inflammatory mediators in the BALF to determine the effect of these cytokine-encoding vectors. Both leukotriene B4 (LTB4) and prostaglandin E2 (PGE2) are potent pro- inflammatory mediator, which are involved in several inflammatory diseases [26]. LTB4 is a potent neutrophil chemoattractant that enhances neutrophil-endothelial interactions and stimulates neutrophil activation [48]. LTB4 may contribute to airway narrowing by producing local edema and increasing mucus secretion. The overproduction of LTB4 plays an important role in the pathogenesis of asthma and acute lung injury [49]. On the other hand, the general consensus is that PGs, in particular PGE2, act to shift the immune response toward a type 2 cytokine profile [50,51]. Moreover, this lipid mediator can also up-regulate IgE production, and consequently support the development of type 2 cytokine- associated inflammatory disorder. However, there is evidence for a broncho- protective role for PGE2 in asthma [52,53]. In recent study, Harizi et al. [54] also demonstrated that PGE2 could inhibit the production of LTB4 from dendritic cells via IL-10 dependent mechanism. It is not clear whether PGE2 synthesis in airway diseases can play a deleterious or a beneficial role. The result of our study indicates that IL-10 can significantly decrease the level of LTB4 in the BALF, but other cytokine gene plasmids may not show a similar effect (Fig. 5). In contrast, all the cytokines together do not have any effect on the level of PGE2 in the BALF (data not shown). Our results may support that IL-10 could inhibit the recruitment of neutrophils by reducing the production of LTB4 (Fig. 3).

Further, we have examined varying doses of IL-10 gene plasmid to test the therapeutic efficiency of this asthma animal model (Fig. 6). It has been reported that instillation of IL-10 protein into the lung could suppress eosinophilic inflammation [55,56]. However, the half-lives of recombinant cytokines are very short and the suppressive effect is weak. In current study, in vivo IL-10 gene delivery can efficiently suppress airway eosinophilia (Fig. 3). In addition, we also demonstrated that IL-10 gene plasmid had a strong immuno-modulating effect in the suppressing Ag-induced eosinophilic inflammation and AHR in a dose-dependent manner (Fig. 6).

As IL-10 is a multi-potent immunosuppressive cytokine, it could exhibit various effects on many resident cells and inflammatory cells, such as endothelial cells, monocytes/macrophages, lymphocytes, and mast cells [57,58]. It has been also reported that IL-10 can inhibits Th1cytokine production via the suppression of IL-12 synthesis in accessory cells [30]. Thus, we combined 2 plasmids (IL-10 and IL-12) to treat OVA-sensitized mice (Fig. 7). Our results showed that in vivo delivery of IL-10 or IL-12 gene alone could efficiently inhibit AHR and airway eosinophilic inflammation. However, combination of IL-10 and IL-12 gene therapy did not exert the syngenic effect in the modulating airway inflammation. It may imply that IL-10 and IL-12 have some antagonistic effect in the local inflammatory site. In addition, the regulating mechanisms of IL-10 and IL-12 for the suppression of experimental asthma are totally different. In the recent study of Nakagome K et al., they pointed that IL-10 gene delivery can suppress lung APC functions and the subsequent Th2 response [59]. IL-10 could reduce the recruitment of eosinophils into the lung by modulating the expression of VCAM-1 on endothelial cells [59] and decreasing the secreting level of eotaxin (in this paper). Thus, IL-10 gene delivery could be more effective in the treatment of local airway inflammation.

Collectively, these data suggests that immunosuppressive cytokines, such as TGF-β and IL-10, as well as Th1-related IL-12, can alleviate the symptom of airway inflammation in a murine model of asthma. Table 1 summarizes the recent studies about the effects of different cytokine in the treatment of asthma. However, the therapeutic mechanisms of these cytokine gene plasmids are different and affect different inflammatory mediators. Further investigation is still needed to analyze the further detailed mechanisms.

**Table 1:** Cytokine directed therapies for asthma

**Treatment**	**Effects**			**Ref.**
	Suppress AHR	Reduce eosinophilia	Other effects	

IFN-γ	Yes	Yes	a. IFN-γ producing T cells increased; b. Reduced IL-4 & IL-5 secretion	60
IL-10	Yes	Yes	a. Down-regulating synthesis of both Th1- & Th2- cytokines b. Reduced both eosinophilia & neutrophilia	59
IL-12	Yes	Yes	a. Th1 skewed response; b. specific IgE/IgG reduced;	5, 9, 11
IL-18	Yes	Yes	Combined IL-12 and IL-18 induce IFN-γ release	6
TGF-β	Yes	Yes	Immuno- regulation, wound healing, and to shorten the inflammatory response	13, 21

## Conclusion

Intra-tracheal administration of TGF-β, IL-10 or IL-12 gene plasmids can efficiently inhibit antigen-induced airway hyper-responsiveness and significantly lower the number of eosinophils and neutrophils in bronchoalveolar lavage fluid of ovalbumin (OVA) sensitized mice during the effector phase. Our data demonstrated that anti- inflammatory cytokines, particularly IL-10, have the therapeutic potential for the alleviation of airway inflammation in murine model of asthma.

## Competing interests

The author(s) declare that they have no competing interests.

## Authors' contributions

CLF prepared plasmids and lipid-plasmid DNA complexes, administrated cytokine plasmid into allergen-sensitized mice, performed AHR, analyzed BAL fluid, did the ELISA, drafted the manuscript, and participated in the design of the study. YLL constructed the single chain IL-12 DNA plasmid. YLY, YLL, and BLC conceived the study and helped to draft the manuscript. All authors read and approved the manuscript.
